# Potential risk factors for reduced quality of life and increased health care utilization in ARDS survivors: results from the multicenter cohort study DACAPO

**DOI:** 10.1186/s13054-024-04992-2

**Published:** 2024-06-19

**Authors:** Hermann Szymczak, Susanne Brandstetter, Sebastian Blecha, Frank Dodoo-Schittko, Magdalena Rohr, Thomas Bein, Christian Apfelbacher

**Affiliations:** 1 Medical Faculty, Institute of Social Medicine and Health Systems Research, Otto von Guericke University Magdeburg, Leipziger Str. 44, 39120 Magdeburg, Germany; 2 University Children’s Hospital Regensburg (KUNO), University of Regensburg, Hospital St. Hedwig of the Order of St. John, Steinmetzstrasse 1-3, 93051 Regensburg, Germany; 3 Medical Sociology, Institute for Epidemiology and Preventive Medicine, University of Regensburg, Franz-Josef-Strauß-Allee 11, 93053 Regensburg, Germany; 4 Department for Anesthesiology, University Hospital Regensburg, Franz-Josef-Strauß-Allee 11, 93053 Regensburg, Germany; 5 Medical Faculty, University of Regensburg, University Hospital Regensburg, Franz-Josef-Strauß-Allee 11, 93053 Regensburg, Germany

**Keywords:** ARDS, Critical illness, Health care utilization, Health related quality of life, Prospective cohort study

## Abstract

**Aim:**

To analyze the association of individual pre-ICU risk factors (obesity, physical and mental comorbidity, smoking status) on the long-term recovery process in survivors of the acute respiratory distress syndrome (ARDS; outcomes: health related quality of life, health care utilization; measured at 12, 24, and 36 months after ICU discharge).

**Findings:**

Results show a possible causal link between pre-ICU risk factors and subsequent recovery of survivors of ARDS, especially with regard to mental health related quality of life.

**Purpose:**

Identifying relevant pre-existing risk factors, such as mental health problems, will enable the identification of at-risk patients, thus aiding in the improvement of long-term healthcare for survivors of critical illness.

## Introduction

Acute respiratory distress syndrome (ARDS) is a common medical condition in critical care medicine, with a prevalence of about 10% in all ICU admissions [1]. Due to a decrease in (in-hospital) mortality during the last decades from about 70–40% [2], interest in the development of long-term outcomes of ARDS survivors, e.g. health related quality of life (HRQoL), has increased [3, 4].

However, little is known about the influence of pre-ICU risk-factors (RF) on the subsequent recovery of ARDS survivors. The identification of relevant pre-existing risk factors (e.g. diminished functional abilities, mental health problems) will enable the identification of patients at risk, and therefore help to improve long-term health care for survivors of critical illness. For instance, early identification of ICU survivors at risk for an adverse post-ICU recovery process might enable medical professionals to specifically treat those patients (e.g. by assigning patients to structured recovery programs), and thereby decreasing societal cost and individual burden of patients and caretakers [5–9].

In the present study, we investigated whether behavioral and medical risk factors at ICU admission might be causally linked to worse outcomes (HRQoL, health care utilization [HCU]) after ICU discharge.

## Materials and methods

A prospective multicenter patient-cohort study with N = 877 adult ARDS survivors from 61 different ICUs across Germany was conducted from 2014 to 2019 (DACAPO [10]). After discharge from hospital, patients received questionnaires at five follow-ups (3, 6, 12, 24, and 36 months after discharge). Methods DACAPO are reported elsewhere (e.g. [11]). The Ethics Committee of the University of Regensburg approved the study (file number: 13-101-0262).

In total, N = 587 participants returned at least one valid questionnaire and were included in the study sample (68% male, age: M [SD] = 53.9 [15.4] years; this distribution is in accordance with other large ARDS cohorts [12]). For the present study, we analyzed data collected at ICU admission (T0), and 12 (N = 396), 24 (N = 218), and 36 (N = 202) months after discharge.

The following pre-ICU risk factors were considered for the computation of a general risk factor score (RF):

- body mass index (BMI) > 30 (i.e. obesity; [13])
- physical comorbidity, based on the *SAPS-II* (Simplified Acute Physiology Score-II [14]), if at least one of seven pre-specified comorbidities was present (active cancer treatment, metastatic cancer, hematologic malignancy, chronic heart failure, AIDS, cirrhosis of the liver, alcohol disorder)
- mental comorbidity, if at least one of four medically diagnosed conditions was present at any time in patients’ life (depression, anxiety-/panic disorder, obsessive compulsive disorder, post-traumatic-stress-disorder [PTSD])
- smoking status, if participants reported to have smoked regularly previously or were active smokers

BMI and physical comorbidity were assessed at ICU-admission. Mental comorbidity and smoking were assessed retrospectively 3 months after ICU discharge via self-report. To compute a general RF score, the presence of every risk factor (yes[1]/no[0]) were summed up for each participant (range: 0–4).

The main outcomes, HRQoL and HCU, are indicators of recovery and were assessed at 12, 24, and 36 months after discharge [15, 16]). HRQoL was assessed via the Physical and Mental Component Scale (PCS, MCS) of the 12-Item Short Form Health Survey (SF-12) [17]. Scores range from 0 to 100 and are standardized for the general German population (M = 50, SD = 10). To assess HCU, participants stated at each follow-up whether and how often they visited any of 13 ambulatory medical specialties during the past 12 months. As a HCU measure, total number of yearly visits (across specialties) was then computed [15].

On a descriptive level, mean (M) and standard deviation (SD) or median (Md) and interquartile range (IQR) are reported for continuous variables, counts and percentages for categorical variables. To ascertain the relationship between RF and HRQoL, bivariate correlations were computed. Subsequently, to assess the unique influence of RF on HRQoL, multiple linear regressions were computed with age, sex and socioeconomic status (SES; assessed at ICU admission via education and occupational level [18]), as control variables. Since HCU data was overdispersed and heavily skewed count data, multivariate negative binomial regression was used to examine the influence of RF, with age, sex and socioeconomic status as control variables.

## Results

Descriptively, PCS and MCS remained stable across measurement points, albeit below the population norm of Md = 50 [17] (PCS-12: Md[IQR] = 42–43[17–20]; MCS-12: Md[IQR] = 45–47[24–25]; cf. [16]). Similarly, HCU remained fairly stable across measurement points (Md[IQR] = 14–16[13–16] visits during the past 12 months), but markedly elevated when compared with the German population [15]).

An overview of RF is provided in Table 1. About one third of patients were obese (BMI > 30) at ICU-admission, one fifth had at least one physical comorbidity, and one fifth at least one mental comorbidity. About 60% of our sample stated to have smoked regularly at one point in their lives or were current smokers. The resulting RF score shows that 19% of participants had no risk factor present at ICU admission, whereas the majority had one or two risk factors present (37% and 33%).

**Table 1:** Individual pre-ICU risk factors and risk factor score (T0)

Variable	Valid (*N*)	Summary	Risk factor yes; n (%)
*Individual risk factors*			
BMI^1^	561	Md (IQR) = 28 (24–32)	183 (32.6)
Physical comorbidity^2^	584	0 n = 466 (79.8%)	118 (20.2)
		1 n = 106 (18.2%)	
		2 n = 12 (2.0%)	
Mental comorbidity^3^	429	0 n = 343 (80.0%)	86 (20.0)
		1 n = 39 (9.1%)	
		2 n = 34 (7.9%)	
		3 n = 9 (2.1%)	
		4 n = 4 (0.9%)	
Smoking status^4^	425	Never smoked n = 154 (36.2%)	271 (63.8)
		Currently smoking n = 54 (12.7%)	
		Quit smoking n = 217 (51.1%)	
*Risk factor score (RF)*			
	401	0 n = 74 (19%)	
		1 n = 148 (37%)	
		2 n = 134 (33%)	
		3 n = 41 (10%)	
		4 n = 4 (1%)	
		M (SD) = 1.4 (0.9)	

^1^BMI: Body mass index, assessed at baseline (T0), Risk factor “yes” if BMI > 30 (i.e. obesity, cf. [13]); ^2^Risk factor “yes” if at least one of seven comorbidities, based on Physical Comorbidity assessment via *SAPS-II* (Simplified Acute Physiology Score-II [14]), assessed at baseline (T0); ^3^Risk factor “yes” if at least one of four diagnosed mental disorders, assessed via self-report 3 months after ICU discharge; ^4^(former) smoker; RF: Risk Factor Score (Range 0 [low risk] to 4 [high risk])

### Health related quality of life

Correlations and main results of the regression models are presented in Table 2. Age was negatively associated with PCS at all follow-ups, but not with MCS. SES and RF were consistently associated with HRQoL (all *p*s < 0.05). However, these statistically significant bivariate relationships between RF and MSC/PCS ceased to be significant for PCS at all measurement points (and for MSC at 36 months) when controlling for age, sex, and SES (Table 2*Multiple regression*; RF: β = − 0.161 to − 0.084, *p* values > 0.05), and remained significant for MCS at 12 and 24 months (RF: β = − 0.235 and − 0.317, *p* values < 0.05).

**Table 2:** Correlations and main results for linear multiple regression

		Correlations (r)									Multiple regression^2^: N, β Risk Factor (adjusted for age, sex, SES), R^2^ model		
		2	3	4	5	6	7	8	9	10	N	β (RF)	R^2^
1	Age	**0**.**106**	0.049	0.093	− **0**.**242**	− **0**.**212**	− **0**.**286**	0.063	0.108	0.005			
2	Sex		**0**.**101**	0.032	− **0**.**120**	**0**.**155**	− 0.084	**0**.**196**	**0**.**161**	**0**.**221**			
3	SES^1^			− **0**.**149**	**0**.**164**	**0**.**216**	**0**.**205**	**0**.**118**	**0**.**196**	**0**.**194**			
4	RF^1^				− **0**.**152**	− **0**.**222**	− **0**.**200**	− **0**.**253**	− **0**.**321**	− **0**.**167**			
5	PCS 12 mo.				–	**0**.**784**	**0**.**693**	**0**.**253**	**0**.**289**	**0**.**226**	213	− 0.084	0.105
6	PCS 24 mo.					–	**0**.**786**	**0**.**325**	**0**.**194**	**0**.**287**	128	− 0.161	0.134
7	PCS 36 mo.						–	**0**.**195**	**0**.**196**	**0**.**264**	113	0.091	0.139
8	MCS 12 mo.							–	**0**.**741**	**0**.**671**	213	− **0**.**235**	0.102
9	MCS 24 mo.								–	**0**.**739**	128	− **0**.**317**	0.170
10	MCS 36 mo.									–	113	− 0.095	0.083

Bold printed r/β values are statistically significant (*p* < .05), N correlations = 117 – 587; ^1^at T0. ^2^Multiple Regression Model variables: age (years), sex (0 = female, 1 = male), SES (socioeconomic status, Range 1 [low] to 7 [high]), RF: Risk Factor Score (Range 0 [low risk] to 4 [high risk]). PCS Physical Component Scale SF-12, MCS Mental Component Scale SF-12

### Health care utilization

For HCU, negative binomial regression models were computed with age, sex, SES, and RF as predictors. Age, sex, and SES were not significantly associated with HCU in any model (*p* = 0.135–0.856). Importantly, associations between HCU and RF were not significantly associated in the models for 12 (B = 0.090, *p* = 0.106, McFadden's pseudo R-square for full model = 0.003) and 24 months (B = 0.092, *p* = 0.332, McFadden's pseudo R-square for full model = 0.003). At 36 months, however, HCU was significantly associated with RF (B = 0.272, *p* = 0.001, McFadden’s pseudo R-square for full model = 0.009).

## Discussion

In this study with ARDS survivors, we found statistically significant correlations between risk factors (pre-existing physical and mental co-morbidity, smoking and obesity) and HRQoL at all measurement points, and with HCU at 36 months. However, after controlling for age, sex, and SES, associations only remained significant for *mental* HRQoL at 12 and 24 months. This might be due to (the control variable) age explaining a significant amount of variance in PCS but not MSC (as indicated by the correlations in Table 2). Thus, the putative causal role of our RF score seems specific to the area of mental HRQoL. The early identification of at-risk patients might therefore be especially beneficial regarding mental well-being, and possibly the prevention of mental disorders such as PTSD.

Existing research on pre-ICU risk factors for post-ICU recovery is comparatively sparse. In a recent consensus paper on the prediction of long-term impairments after critical illness, Mikkelsen et al. [9] list the following clinically relevant *preexisting* risk factors for the functional domains of mental and physical health: mental health problems (anxiety, depression, PTSD), physical impairment, frailty, cognitive impairment. Our RF measure also contained preexisting physical and mental comorbidity. As an important addition to previous studies, we also considered behavioral risk factors: Smoking and obesity (both particularly relevant for ARDS patients).

Regarding the prevalence of individual risk factors, it is noteworthy that our sample deviates from the general population, but not substantially: About 56% of the German population have smoked at some point in their lives [19] (vs. 64% in our sample), and about 20% [20] are obese (vs. 33% in our sample). Regarding mental health, about 28% of the German population meet criteria for at least one mental disorder (12-month prevalence), according to the Mental Health Module of the representative German Health Interview and Examination Survey for Adults (DEGS1-MH, [21]), which includes 25 diagnoses, however.

The main strength of this work is that we used data from a large prospective cohort study with multiple time points over a course of four years to ascertain the possible influence of pre-ICU risk-factors on recovery. This approach is valuable as recovery from critical illness is a long-term and dynamic process.

One of the main limitations of the present study is that (pre-ICU) risk-factors were only assessed at ICU admission or retrospectively, three months after hospital discharge. Naturally, the prospective assessment of risk factors before the development of ARDS would have been more valid. However, one of the main methodological challenges for prospective cohort studies on ARDS survivors is the assessment of pre-ICU factors. Even though this information would be important in order to ascertain the actual (and unique) influence of critical illness survival on the lives of patients it is not available in existing patient cohorts which start recruitment only with the occurrence of critical illness.

In sum, our results showed a possible causal link between pre-ICU risk factors and subsequent recovery of survivors of ARDS, especially with regard to mental HRQoL. Future research might analyze whether further risk factors, e.g. high blood pressure, may have a stronger influence. Also, possible protective factors, e.g. social support [5], should be analyzed.

## Data Availability

The datasets used and/or analyzed during the current study are available from CA on reasonable request.

## Abbreviations

ARDS: Acute respiratory distress syndrome
DACAPO: Surviving ARDS: the influence and quality of care and individual patient characteristics on health-related quality of life (DACAPO study)
DEGS: German Health Interview and Examination Survey for Adults
HCU: Health care utilization
HRQoL: Health-related quality of life
ICU: Intensive Care Unit
IQR: Interquartile Range
M: Mean
Md: Median
N: Sample size
MCS-12: Mental Component Summary of Health Related Quality of Life
PCS-12: Physical Component Summary of Health Related Quality of Life
RF: Risk factor(s)
SAPS-II: Simplified acute physiology score-II
SD: Standard deviation
SES: Socio economic status
SF-12: Short form 12 survey (Health Related Quality of Life)
